# In Situ Ag-MOF Growth on Pre-Grafted Zwitterions Imparts Outstanding Antifouling Properties to Forward Osmosis Membranes

**DOI:** 10.1021/acsami.0c12141

**Published:** 2020-07-17

**Authors:** Mehdi Pejman, Mostafa Dadashi Firouzjaei, Sadegh Aghapour Aktij, Parnab Das, Ehsan Zolghadr, Hesam Jafarian, Ahmad Arabi Shamsabadi, Mark Elliott, Mohtada Sadrzadeh, Marco Sangermano, Ahmad Rahimpour, Alberto Tiraferri

**Affiliations:** † Department of Environment, Land and Infrastructure Engineering (DIATI), Politecnico di Torino, Corso Duca degli Abruzzi 24, 10129 Turin, Italy; ‡ Department of Civil, Construction and Environmental Engineering, University of Alabama, Tuscaloosa, Alabama 35487, United States; § Department of Mechanical Engineering, 10-367 Donadeo Innovation Center for Engineering, Advanced Water Research Lab (AWRL), University of Alberta, Edmonton, AB T6G 1H9, Canada; ∥ Department of Chemical & Materials Engineering, University of Alberta, Edmonton, AB T6G 1H9, Canada; ⊥ Department of Physics and Astronomy, University of Alabama, Tuscaloosa, Alabama 35487, United States; # Department of Mining and Metallurgical Engineering, Amirkabir University of Technology, Tehran 159163-4311, Iran; ∇ Department of Chemistry, University of Pennsylvania, Philadelphia, Pennsylvania 19104, United States; ○ Department of Applied Science and Technology, Politecnico di Torino, Corso Duca Degli Abruzzi 24, 10129 Turin, Italy; ◆ Department of Chemical Engineering, Babol Noshirvani University of Technology, Shariati Avenue, Babol Mazandaran, 4714871167, Iran

**Keywords:** TFC membranes, biofouling, metal−organic frameworks, forward osmosis, zwitterions, antifouling

## Abstract

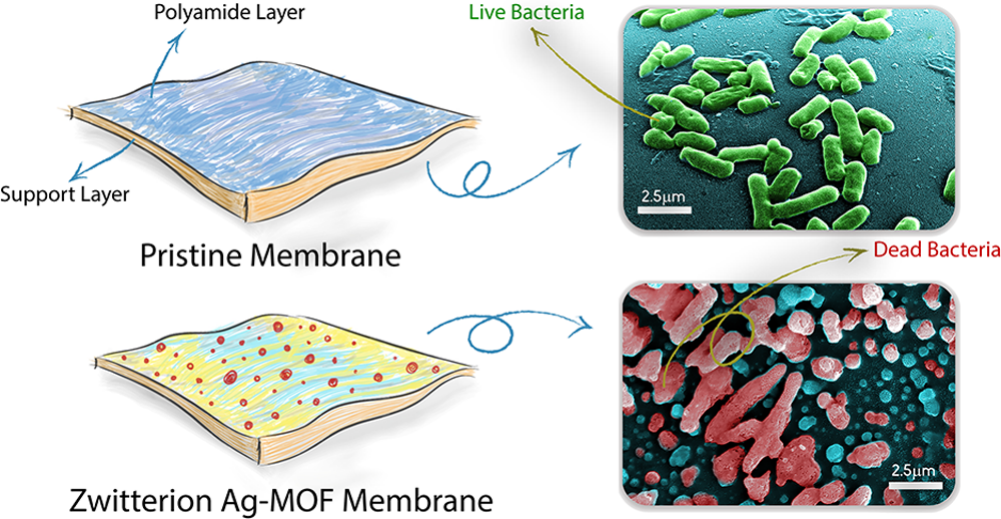

In this study, a polyamide forward osmosis membrane was functionalized with zwitterions followed by the in situ growth of metal–organic frameworks with silver as a metal core (Ag-MOFs) to improve its antibacterial and antifouling activity. First, 3-bromopropionic acid was grafted onto the membrane surface after its activation with *N*,*N*-diethylethylenediamine. Then, the in situ growth of Ag-MOFs was achieved by a simple membrane immersion sequentially in a silver nitrate solution and in a ligand solution (2-methylimidazole), exploiting the underlying zwitterions as binding sites for the metal. The successful membrane functionalization and the enhanced surface wettability were verified through an array of characterization techniques. When evaluated in forward osmosis tests, the modified membranes exhibited high performance and improved permeability compared to pristine membranes. Static antibacterial experiments, evaluated by confocal microscopy and colony-forming unit plate count, resulted in a 77% increase in the bacterial inhibition rate due to the activity of the Ag-MOFs. Microscopy micrographs of the *Escherichia coli* bacteria suggested the deterioration of the biological cells. The antifouling properties of the functionalized membranes translated into a significantly lower flux decline in forward osmosis filtrations. These modified surfaces displayed negligible depletion of silver ions over 30 days, confirming the stable immobilization of Ag-MOFs on their surface.

## Introduction

As an emerging technology, forward osmosis (FO) is attracting significant interest for numerous applications, such as desalination and wastewater treatment.^1−3^ Although its fouling behavior is considered better than that of pressure-driven processes,^4^ fouling and biofouling remain limiting factors to the effective implementation of FO.^5^ Fouling of thin-film composite (TFC) polyamide (PA) membranes is a direct function of the structural and chemical properties of the membrane surface, including roughness, surface charge, wettability, and the presence and nature of functional groups.^6,7^ Among all forms of fouling, biofouling seems to be the most challenging one.^1^ Biofouling is initiated by microbial deposition onto the membrane surface, followed by cell proliferation and production of extracellular polymeric substances. These substances provide an appropriate environment for the life of microorganisms and the ensuing biofilm formation.^8,9^ Biofilm and fouling layers in general hinder water permeation and increase the energy consumption of the process. Even trace amounts of microorganisms can produce significant biofouling on the membrane surface.^10^

One approach toward biofouling mitigation is the surface modification of the membrane through the anchoring of biocidal agents.^11^ The application of silver-based materials has been reported to provide membranes with “attacking” anti-biofouling properties due to the broad and strong antimicrobial activity of silver against fungi, bacteria, and viruses.^12^ However, some concerns always exist with the immobilization of silver nanomaterials, including silver leaching due to little resistance to washing,^13,14^ as well as incompatibility issues between inorganic materials and the organic membrane matrix, which can create defects and deteriorate membrane selectivity.^15^

Metal–organic frameworks (MOFs) are coordinated polymers composed of organic linkers and metal cores.^16−18^ The key advantage of MOFs over fully inorganic structures is their tunability through the choice of various metals and/or organic linkers.^19,20^ This tunability has the advantage of allowing the construction of MOFs with better compatibility with the polyamide layer. Also, the ion release issue is more easily controlled by the adjustment of the organic frame, which can act as a barrier against high rates of metal leaching.^21,22^ MOFs not only provide a reservoir of uniformly distributed biocidal metal ions on a membrane surface but also prevent aggregation of nanomaterials, thereby leading to sustained antimicrobial activity.^23^ An additionally attractive approach is represented by the coupling of the antibacterial property of Ag-based MOFs with the “defensive” antifouling activity provided by zwitterions.^24,25^ The zwitterionic architecture adds a strong binding interaction for water molecules, which facilitates the formation of a hydration layer on the active surface of the membrane and, hence, a lowered fouling propensity.^26,27^

This work proposes a simple procedure to achieve functionalization of a polyamide membrane with combined active biocidal Ag-MOFs and passive anti-adhesion hydrophilic zwitterions. To achieve this goal, carboxyl-rich zwitterions are first grafted onto the polyamide, and then, a simple dipping procedure is adopted to promote the effective in situ growth of Ag-MOFs. The membranes are fully characterized to confirm the success of the functionalization strategy and to investigate their newly obtained chemical, physical, and morphological properties. The impact of functionalization on the water permeability and salt selectivity is evaluated in FO experiments. The antibacterial activity of functionalized membranes is assessed through confocal microscopy and plate counts, while the rate of silver release from the membranes is quantified. Ultimately, the antifouling/anti-biofouling capability of the membranes is discussed based on their flux behavior in medium-term filtration experiments.

## Experimental Section

### Materials

2-Methylimidazole (2MI) was used as an organic ligand and silver nitrate (AgNO_3_) as a metal source for the synthesis of Ag-MOFs. Potassium persulfate, *N*,*N*-diethylethylenediamine (DEDA), and sodium metabisulfite were used to prepare the solution to graft zwitterions onto the polyamide membrane. The carboxyl-based zwitterion was bromopropionic acid (BPA). Sodium hydroxide and nitric acid were added to the solutions for pH adjustment. Sodium chloride (NaCl) was used as a draw solute in the FO filtration experiments. TFC membranes were used as pristine membranes and soaked in 1 wt % sodium metabisulfite for one day before use. The membranes are composed of a polyethersulfone support layer and a fully aromatic polyamide active layer, without any additional surface coating. The transport parameters of the membranes, determined by FO experiments, are pure water permeance, *A*, equal to 2.26 L m^–2^ h^–1^ bar^–1^; NaCl permeability coefficient, *B*, equal to 0.94 L m^–2^ h^–1^; and structural parameter, *S*, equal to 337 μm.

### Preparation of the Ag-MOFs When Not Grown In Situ

Ag-MOFs were prepared following a procedure described in our previous publication.^28^ The metal solution was prepared by adding 0.6 g of AgNO_3_ in 90 mL of deionized (DI) water; the ligand solution was obtained using a 90 mL ethanol solution containing 2MI (1.05 g). The two solutions were stirred for 15 min and sonicated for 2 min, respectively. Then, the 2MI solution was gently poured into the metal solution, and the resulting mixture was stirred for 30 min. The suspension was kept stagnant for 3 h to precipitate the formed Ag-MOFs and facilitate removal of the supernatant. The precipitate was washed to remove any unreacted substances. Next, it was centrifuged twice using ethanol as a rinsing solvent. Finally, the powder was dried for 18 h at 60 °C.

### Membrane Functionalization

First, an activation solution (pH 5) containing potassium persulfate (0.03 wt %), DEDA (2 wt %), and sodium metabisulfite (0.02 wt %) was gently poured onto the rubber-framed membrane (pristine membrane, labeled as “M0”) at room temperature. This solution was left to sit for 1 h, promoting the amidation of DEDA molecules with the carboxyl groups of the membrane surface.^29^ DEDA-grafted membranes were subject to two different fabrication methods. Membranes labeled as “M1” were obtained by covering the DEDA-grafted surfaces with a BPA aqueous solution (5 wt %, pH 5.0), at 40 °C for 20 h, to graft COO^–^-rich zwitterions. Membranes labeled as “M2” were instead obtained by covering the DEDA-grafted surfaces with a solution containing both BPA (5 wt %, pH 5.0) and Ag-MOFs (0.05 wt %). In the latter case, the use of a mixture of *N*-methyl-2-pyrrolidone (NMP, 10 wt %) and water (90 wt %) as a solvent was necessary to achieve a suitable dispersion of Ag-MOFs. For the in situ formation of Ag-MOFs, a metal solution (1.5 wt % in 180 mL of DI) and a ligand solution (2.5 g in 160 mL of ethanol) were separately prepared. The membranes, M1 and M2, were then both immersed into the metal solution followed by the ligand solution, each time for 30 min. The samples were subsequently heat-cured at 50 °C for 1 h. Based on these procedures, the more streamlined functionalization of M1 membranes entailed the surface modification by Ag-MOFs solely via in situ growth. On the other hand, Ag-MOFs were also pre-deposited on the surface of M2 membranes, following a more traditional and consolidated approach, albeit more involved.

**Figure 1:**
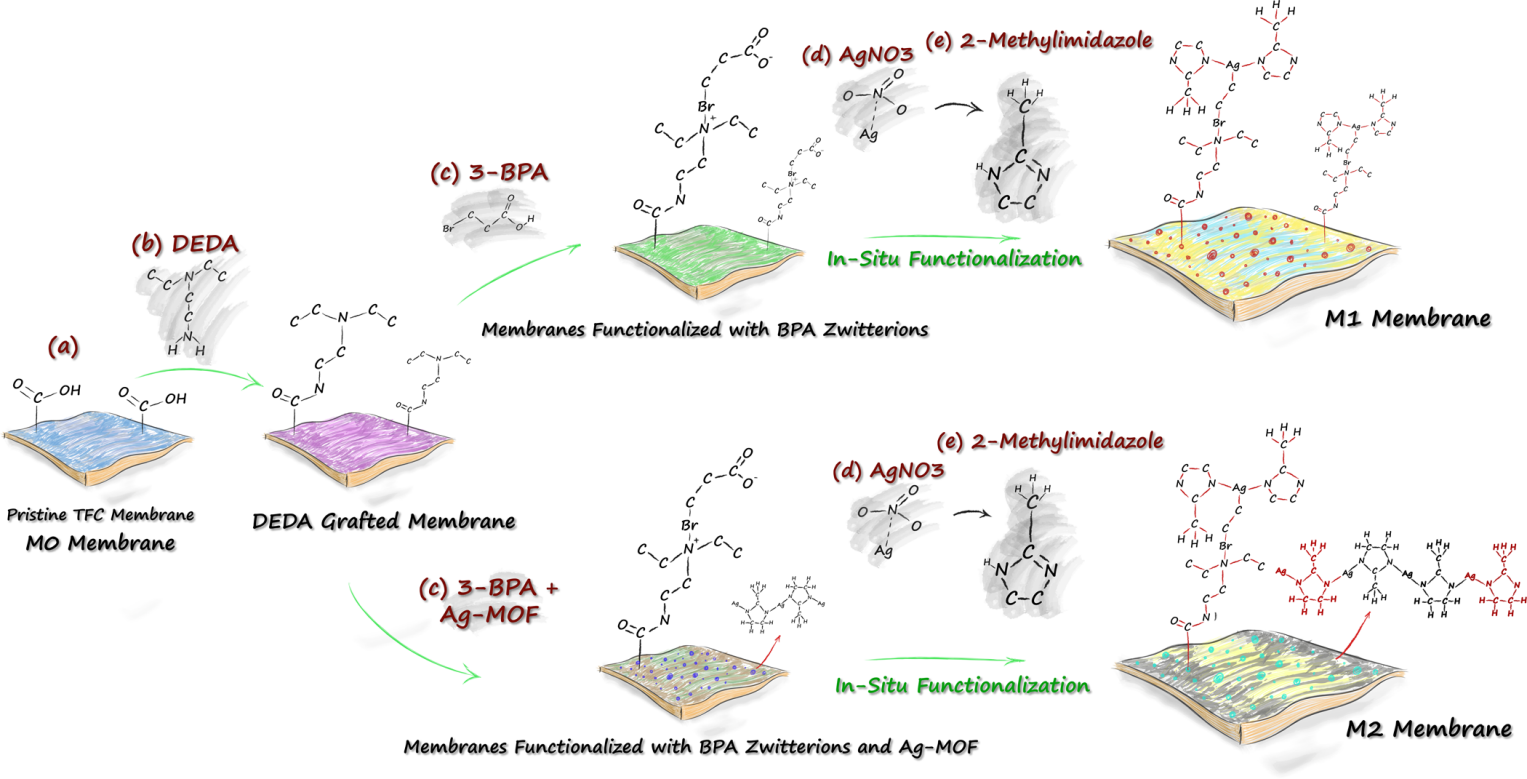
Illustrative scheme of the steps involved in the preparation of ZW-Ag-2MI nanocomposites starting from (a) pristine polyamide membranes (M0) through (b) immersion in the DEDA solution and subsequently (c) grafting of ZW structures and in situ growth of silver-rich MOFs by (d) deposition of Ag^+^ ions (yellow) via immersion in a AgNO_3_ solution followed by (e) immersion in the 2-methylimidazole ligand solution.

Figure 1 depicts the step-by-step functionalization procedure of the membranes. Zwitterions can act as nanoreactor substrates for metal/ligand nanoparticles and immobilize metal ions due to the presence of the negative functional groups.^25,30^ Abundant residual carboxyl functional groups present on the BPA-modified surface can thus act as active sites and promote nucleation of Ag^+^ ions.^31^ Upon silver nucleation, the 2-MI ligand was applied to coordinate with Ag^+^ ions and to self-assemble networks of Ag-2MI MOFs.^11^ Since Ag-2MI contains available nitrogen for metal coordination within the imidazole, these structures are capable of interacting with Ag^+^ ions and facilitating the formation of highly stable Ag-2MI MOFs on the zwitterionic layer.^11,30,32^

### Characterization of the Membranes

The membranes were characterized with attenuated total reflection Fourier transform infrared spectroscopy (ATR-FTIR) and X-ray photoelectron spectroscopy (XPS) to study the chemical properties and to identify the functional groups at the active surfaces. The instrument for ATR-FTIR was a Nicolet iS50 FT (Thermo Fisher Scientific, USA), and the scan range was 500–4000 cm^–1^. The instrument for XPS was a Kratos spectrometer (Axis 165 XPS/Auger, Shimadzu, Japan) equipped with a 100 μm monochromatic Al K(alpha) X-ray. Scanning electron microscopy (SEM; JEOL 7000, JEOL, USA) coupled with energy-dispersive X-ray spectroscopy (EDX; JEOL 7000, JEOL, USA) was employed to observe the surface morphology of the membranes. The membrane samples were sputter-coated with 5 nm of gold (Leica EM ACE600, USA) before the measurements. The roughness of the membrane surface was investigated with atomic force microscopy (AFM; EasyScan II, Switzerland): the average roughness, *R*_a_, and the root-mean-square roughness, *R*_RMS_, were thus calculated for the different samples.^33^ Water contact angles (DSA 100, KRÜSS, Germany) were measured by placing small droplets on five random spots on the samples and evaluating their wettability. Further information on the characterization methods can be found in our previous publications.^28,34,35^

The crystalline patterns corresponding to Ag-MOFs were studied with X-ray powder diffraction (XRD) using a diffractometer (Bruker D8, Germany) equipped with a Cu Kα radiation in 2θ mode from 0 to 60°. A SurPASS Electrokinetic streaming potential analyzer (Anton Paar, Graz, Austria) was adopted to determine the zeta potential of the active surfaces across a pH range of 3–11. The zeta potential measurements were performed at 25 °C in a background electrolyte solution composed of 1 mM KCl and using HCl and NaOH as acid and base for pH adjustment, respectively.

### Evaluation of the Transport Parameters of the Membranes

The filtration transport properties of the membranes, including the water flux (*J*_w_), the reverse solute flux (*J*_s_), and *J*_w_/*J*_s_ ratios, were determined with a cross-flow FO unit able to allocate a membrane sample with a net surface area of 30 cm^2^ and described in our previous publication.^28^ Briefly, the system comprises pumps to circulate the draw solution (DS) and the feed solution (FS) on the two sides of the membrane and is capable of monitoring the flow using an adjustable flowmeter. The cross-flow velocity was set at 20 cm/s. DI water and NaCl solutions (0.5, 1, 1.5, 2 M) were used as the FS and DS, respectively. The transport parameters were obtained using the method proposed by Tiraferri et al.^36^

### Fouling and Biofouling Assessment

Fouling tests were conducted to evaluate the organic fouling and biofouling behavior of the membranes following the procedure described in our previous studies.^18,28,34,37^ Sodium alginate was selected as a model organic foulant and dissolved (250 mg/L) in water to obtain the foulant solution.^38−40^ The experiments were performed starting with feed and draw volumes of 3 L. The permeate flux stabilized at the initial value of 20 ± 1 L m^–2^ h^–1^ using the appropriate concentration of DS prior to the addition of the foulant. The FO test was run for 24 h at a cross-flow velocity of 8.5 cm/s, and the permeate flux was monitored by means of an electronic balance. For the biofouling assessment, the same protocol was followed by using *Escherichia coli* (*E. coli*) bacteria, instead of alginate, at a concentration of 10^7^ CFU/L.

### Antibacterial Activity of the Membranes

*E. coli* was used as a model gram-negative bacterial microorganism to evaluate the antibacterial activity of the synthesized membranes. The *E. coli* was cultured in trypticase soy broth (TSB) by incubating at 37 °C overnight with proper shaking. Freshly prepared TSB was inoculated with the overnight culture of *E. coli* and was again incubated at the same temperature for 3 h. Three methods were adopted to investigate the antibacterial properties of the membranes. Confocal microscopy and heterotrophic plate count were used to determine the relative survival of unattached bacteria that had been exposed to the surfaces of the tested membranes and the survival of attached bacteria on the membrane surfaces, respectively; SEM was used to determine the morphological conditions of bacterial cells.

For heterotrophic plate count experiments, the bacteria culture was centrifuged at 6000 rpm for 3 min, and a bacterial pellet was obtained; the pellet was then re-suspended in sterile phosphate buffer solution (PBS), and a final bacterial solution of 10^7^ CFU/mL concentration was obtained. For each membrane, 1 × 1 cm^2^ of the membrane-active surface was exposed to the bacterial solution (1 mL) in Petri dishes and was incubated at 37 °C for 1 h with shaking. The 1 × 1 cm^2^ samples were then washed with sterile 10 mL of PBS to remove the unattached cells from the membranes. This rinse solution was then placed on the trypticase soy agar plates, and these plates were again incubated for 1 day at 37 °C (without shaking) to determine the viability of the unattached cells. The number of cells was counted in terms of colony-forming units (CFU).

For confocal microscopy tests, a LIVE/DEAD BacLight bacterial viability kit (Thermo Fisher Scientific) was used to determine the viability of the attached cells on the membranes. Propidium iodide (PI) and SYTO 9 were used to investigate the proportion of attached cells that were viable. After bacterial contact, the membranes were stained with PI and SYTO9 and were incubated in the darkroom for 15 min. Then, the membranes were again washed in sterile PBS to get rid of the extra staining material, prior to microscopic analysis. The samples were then mounted on glass slides and examined under a Nikon C2 laser scanning confocal microscope. An EGFP laser at 495–547 nm was employed for SYTO 9, and a TRIC laser at 566–624 nm was used for PI stain excitation. The SYTO 9 caused the viable cells to fluoresce green, while the PI caused the dead cells to fluoresce red. A digital image capture system was used to take 18 images on different spots of each membrane sample to count the total dead and live bacteria. For each viability test, two different membranes were used, and the average is reported.

For the SEM images, the membrane samples exposed to bacteria were washed with 2.5% glutaraldehyde, again washed with PBS and sterile DI water, and then successively washed with different percentages of laboratory-grade ethanol to enable clear imaging of the healthy viable bacterial cells and damaged bacterial cells on the tested membranes. A Thermo Scientific Apreo scanning electron microscope was used for the image capture of the live/dead bacterial cells on the membranes.

### Silver Ion Leaching Test

To evaluate the release rate of silver ions in batch mode, membrane coupons (4 cm^2^) were incubated in 20 mL of DI water under mild shaking followed by acidification using a 1% nitric acid aqueous solution and shaking (100 rpm) for 30 days. Water samples were periodically analyzed after 1, 7, and 30 days using inductively coupled plasma mass spectrometry (ICP-MS; 143 NEXION 300D, PerkinElmer) to determine the leached Ag ion concentration.^37^

## Results and Discussion

### Surface Characteristics of the Membranes

**Figure 2:**
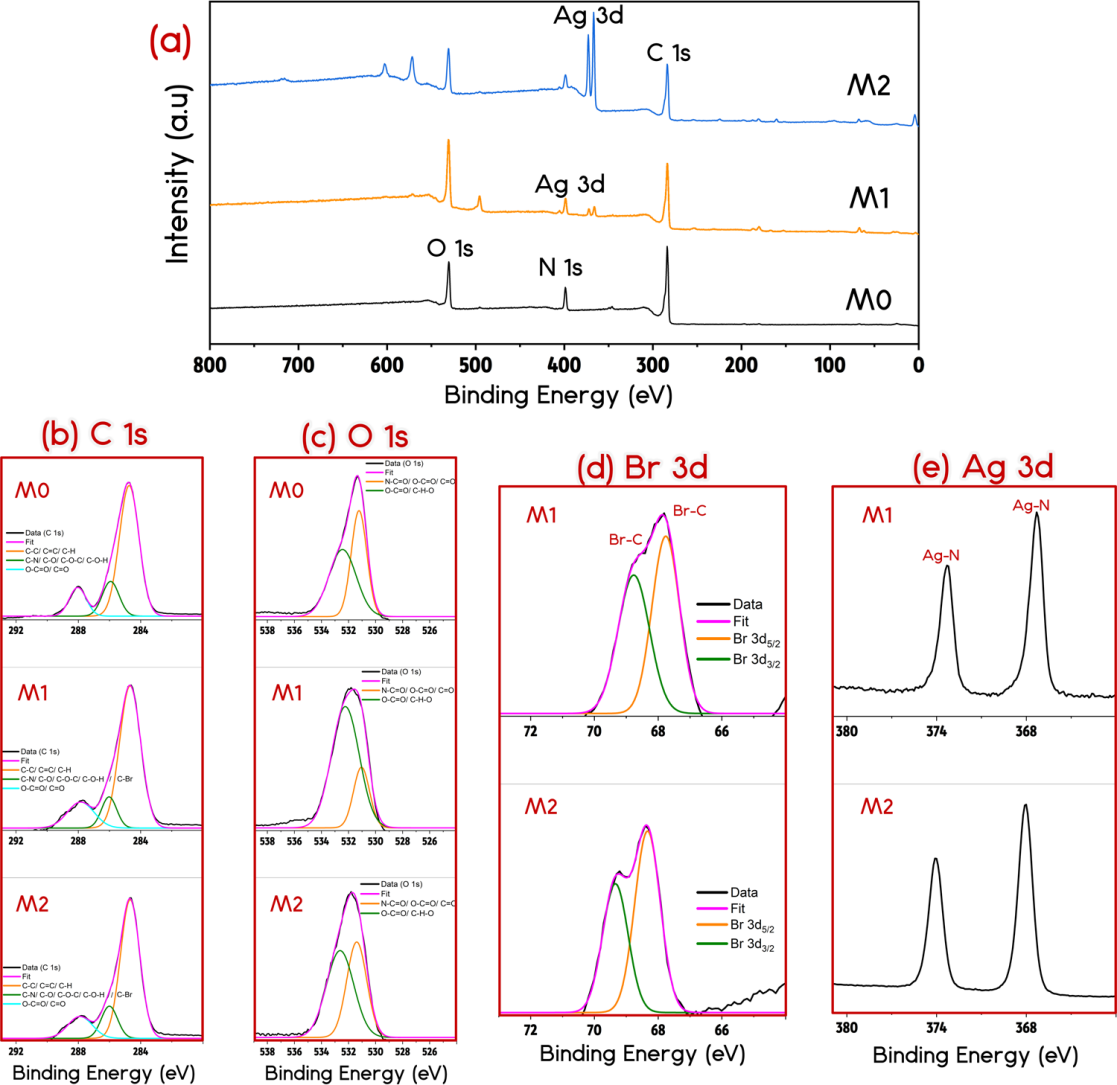
XPS analyses for the pristine and functionalized membranes: (a) entire XPS spectra, fitted (b) C 1s and (c) O 1s regions for the pristine and functionalized membranes, and fitted (d) Br 3d and (e) Ag 3d peaks for the functionalized membranes.

Different characterization techniques were combined to verify the success of the surface functionalization. ATR-FTIR spectroscopy of the membrane surface showed the peaks typically associated with aromatic polyamide and suggested the presence of BPA zwitterions on the modified surface through a characteristic peak associated to C=O stretching of the carboxyl group; see the Supporting Information, Figure S1, for further information. The survey and high-resolution XPS spectra of M0, M1, and M2 membranes are presented in Figure 2. As expected, all membranes showed peaks for carbon (C), nitrogen (N), and oxygen (O). Due to bromine (Br) bonding with carbon atoms, a Br peak centered approximately at 68.5 eV corroborates the presence of the 3-BPA zwitterion on the surface of M1 and M2 membranes. The Br 3d peak deconvolutes to Br 3d_5/2_ and Br 3d_3/2_ bonds, respectively, at 67.7 and 68.7 eV for M1 and 68.3 and 69.3 eV for M2.^41,42^ Moreover, the appearance of Ag 3d_5/2_ and Ag 3d_3/2_ peaks for M1 and M2 are mainly due to silver bonding with nitrogen atoms around 367.5 and 373.5 eV, respectively,^43,44^ supporting the existence of ZW/Ag-MOF nanocomposites in both the functionalized membranes. Clearly, a relatively more intense set of Ag peaks was obtained in M2 compared to M1 due to the different fabrication procedure step of Ag-MOF deposition for M2. Regarding C 1s, the peak centered approximately at 284.6 eV is assigned to C–C, C=C, and C–H bonds; the peak around 286.2 eV is attributed to C–N, C–Br, C–O, C–O–C, and C–O–H bonds; and the peak at approximately 288.5 eV is ascribed to O–C=O and C=O bonds.^37,45−48^ Finally, regarding O 1s, the peak around 531 eV is reportedly attributed to N–C=O, O–C=O, and C=O bonds. O–C=O and C–O–H bonds may be assigned to the peak around 532 eV.^37^ These results strengthen the hypothesis that zwitterion structures are effective in interacting with metal ions.^25,30^ The abundant residual carboxyl moieties of the BPA-modified surface reasonably acted as binding sites for Ag^+^.^31^ The 2-MI ligand was then able to coordinate with Ag^+^ ions and form stable networks of Ag-2MI MOFs on the ZW architecture by in situ growth.^11,30,32^

**Figure 3:**
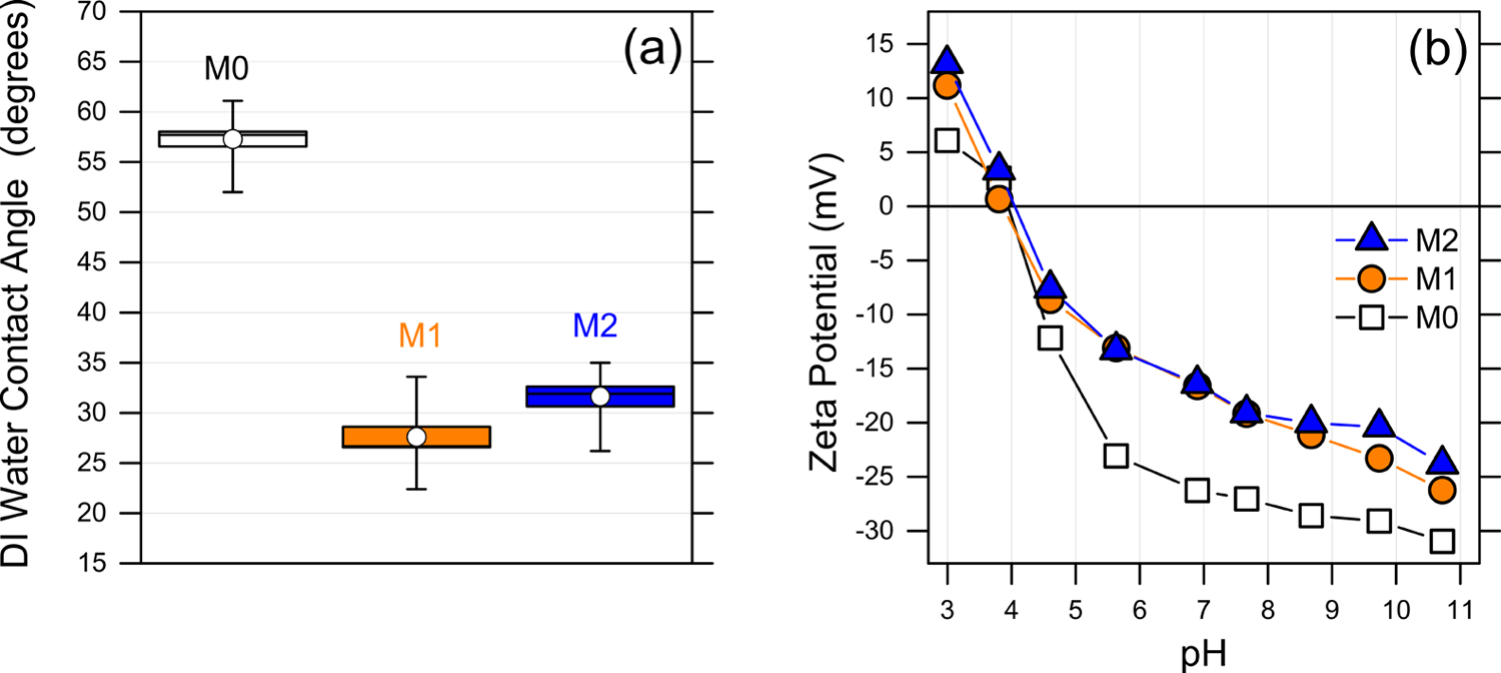
(a) Results of contact angle measurements of DI water on the membranes and (b) zeta potential of the surfaces as a function of pH in 1 mM KCl at room temperature.

Contact angle measurements were performed to assess the degree of relative wettability of the membranes (Figure 3a). The results showed an average angle reduction of roughly 50% for both functionalized membranes (M1 and M2) compared to the pristine membrane (M0), suggesting increased surface wettability as the result of functionalization. This enhanced wettability is mainly ascribed to the moieties of BPA, leading to an increased affinity between water and the membrane surface. The M1 and M2 membrane showed similar average contact angles, within experimental error. The surface zeta potentials are plotted in Figure 3b. The membranes exhibited a negative surface charge for pH values above approximately 4, which is roughly the p*K*_a_ of carboxyl groups.^49^ The partial neutralization of zwitterionic carboxyl groups via amidation caused an upward shift to less negative potential values, equally for M1 and M2. The shift toward more neutral zeta potential may also be partly attributed to the presence of 2-methylimidazole. This organic linker comprises weakly basic imidazole nitrogen, which is exploited to form the complex with silver, but may protonate around pH 7 if not coordinated with the metal. A negative zeta potential near the neutral pH range is useful to repel organic and biological foulants; however, the in situ growth of Ag-MOFs can minimize the amount of exposed carboxyl groups, which can promote fouling through interactions with Ca^2+^ and other multivalent cations.^50,51^

**Figure 4:**
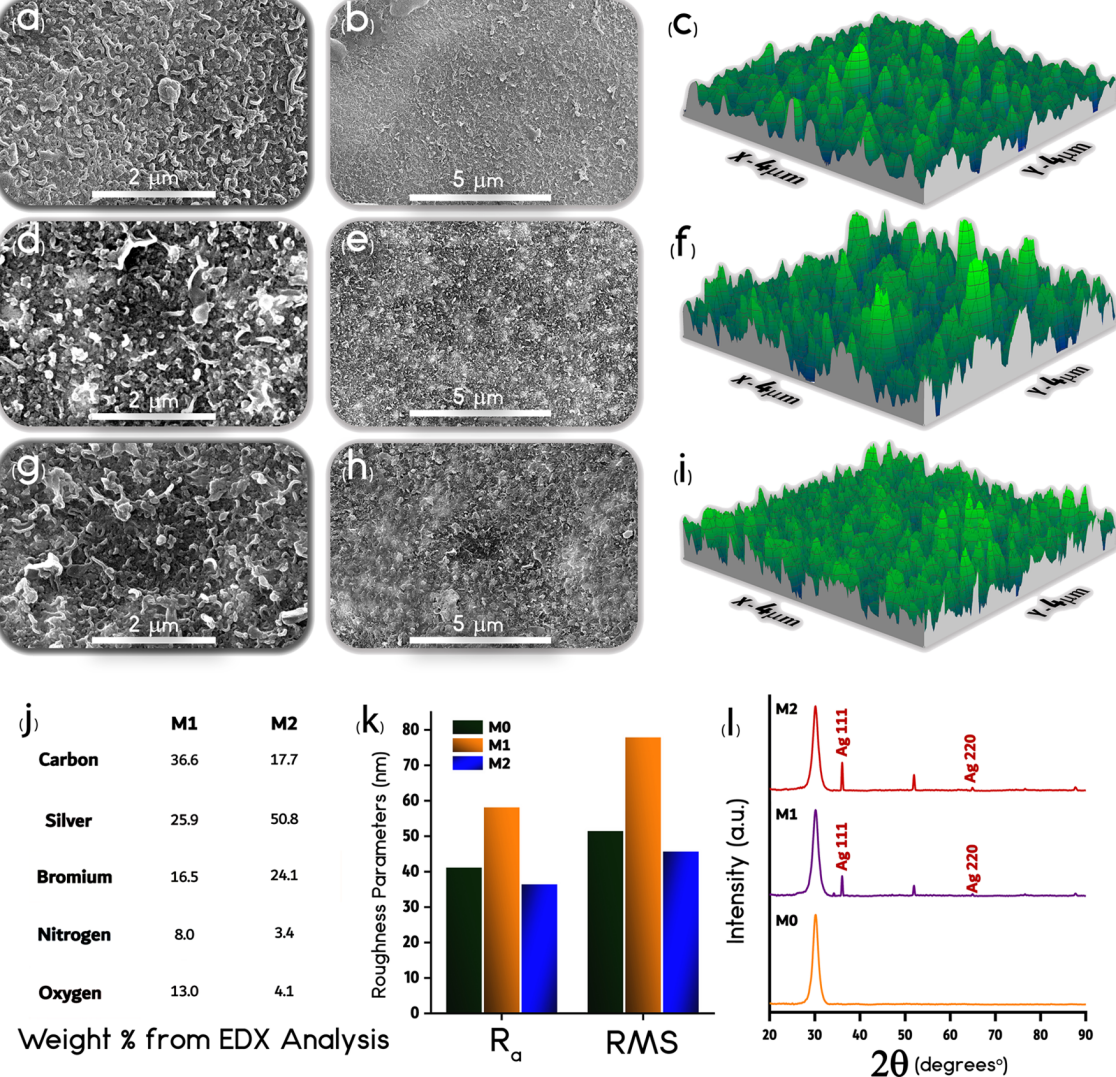
Representative surface SEM micrographs of (a and b) M0, (d and e) M1, and (g and h) M2. 3D AFM scans of (c) M0, (f) M1, and (i) M2. (j) Surface elemental analysis from EDX measurements presented as weight percentage of the main elements. (k) Roughness parameters of all the membranes measured with AFM. (l) XRD patterns of the various membranes (here, the broad peak at 2θ = 30° is attributed to amorphous glass, which was used as the sample holder during the XRD measurements).

Representative SEM micrographs of the pristine membrane (Figure 4a,b) show the typical ridge-and-valley structure of PA membranes,^51^ but additional granule and floc-like structures on the surface of M1 and M2 membranes are apparent (Figure 4d, e, g, and h). These morphologies are consistent with previous observations that the grafting of zwitterions and/or Ag-MOFs could form a thin layer with microscale and nanoscale aggregates on a substrate.^37,52^ The scattered white spots of random distribution on the surface of M1 and M2 membranes can be attributed to the Ag-MOFs. High-magnification micrographs suggest a more pronounced density of white-colored features on M2.^19^ This result is somewhat expected due to the more traditional approach for M2 functionalization. Further morphological insights are provided in terms of roughness parameters, *R*_a_ (average roughness) and *R*_RMS_ (root-mean-square roughness) (Figure 4c, f, and i). M0 and M2 membranes showed close *R*_a_ and *R*_RMS_ values, whereas M1 samples revealed a significant increase in surface roughness (Figure 4i), consistent with observations from SEM results. Possibly, the presence of extra Ag-MOFs in the functionalization solution for the fabrication of the M2 membrane allowed a more homogeneous Ag-MOF immobilization and growth, leading to smoother and more uniform surfaces. An increase in surface roughness is usually associated with a heightened fouling propensity in membranes, but here, there may be a trade-off stemming from the availability of a more hydrophilic surface.^52^ Further observations over grafting of Ag-MOFs and zwitterions performed by the EDX and XRD analyses corroborated the presence of zwitterions and Ag-MOFs on both functionalized membranes (Figure 4j,l). The EDX images displaying elemental distribution and the analysis showing the resulting spectra are provided in the Supporting Information (Figures S2 and S3). The Ag(111) and Ag(220) peaks relative to M1 and M2 membranes corroborate the presence of silver nanoparticles on the modified samples. The presence of peaks at 54.5, 77, and 89° are possibly indicative of the crystalline structure of the coated Ag-MOFs, as suggested by previous studies.^53^ The bromine and silver content found for the samples agrees with the fabrication method, as well as the larger silver content observed in the results relative to M2 compared to M1.^54,55^

Overall, the surface characterizations suggested that both M1 and M2 membranes were successfully functionalized with both zwitterions and Ag-MOFs although the amount and density of these latter structures could not be quantitatively determined. The results from microscopy and EDX mapping indicated that the more traditional approach followed to obtain M2 membranes, comprising a step of Ag-MOF deposition, allowed a more substantial and uniform availability of Ag on the surface. However, even the more innovative and streamlined protocol to achieve M1 membranes, solely based on in situ Ag-MOF growth, resulted in suitable functionalization and surface characteristics in terms of wettability and silver presence.

### Transport Properties of the Membranes

**Figure 5:**
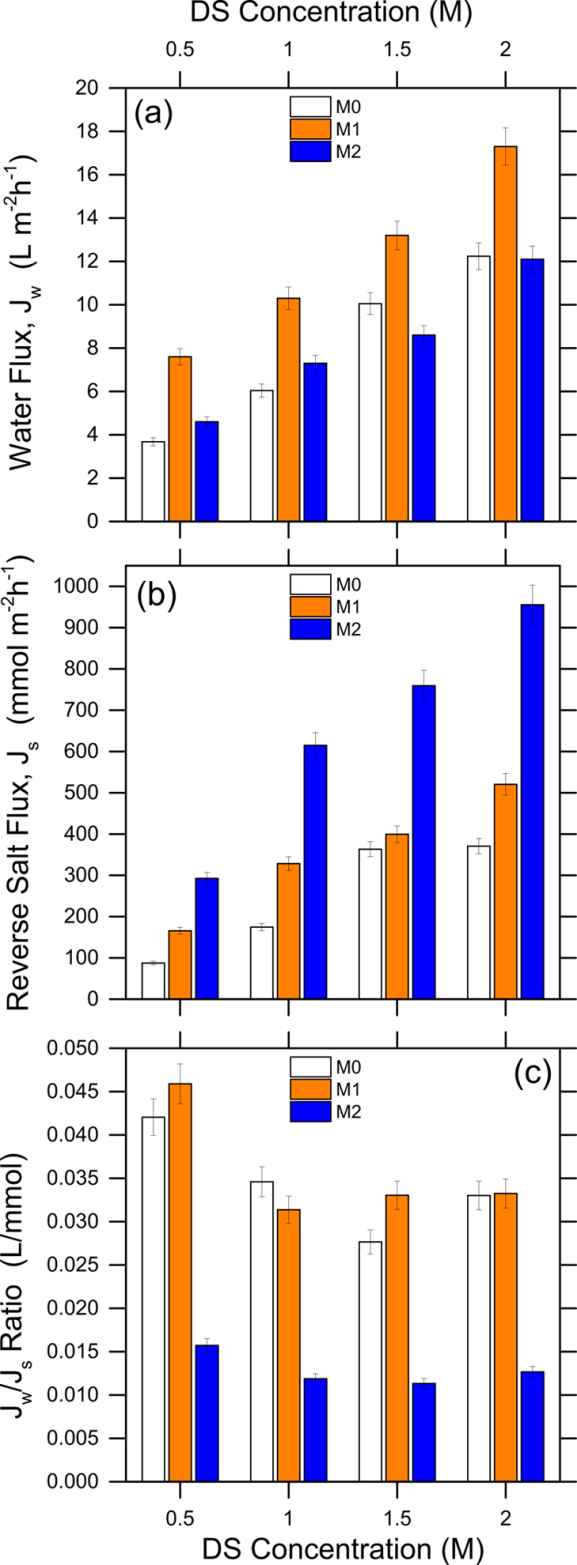
FO transport parameters of pristine and functionalized membranes, measured with a lab filtration setup and in tests using different NaCl concentrations. (a) Permeate flux, *J*_w_, (b) reverse NaCl flux, *J*_s_, and (c) ratio of permeate flux over NaCl flux, *J*_w_/*J*_s_.

FO filtration experiments were performed to investigate the impact of surface functionalization on the transport properties of the membranes (Figure 5a,b). The water flux (*J*_w_) generally increased upon functionalization, which may be attributed to the more hydrophilic surface and the stronger interaction with water molecules for modified membranes. The lower effect observed for M2 may be the result of a higher resistance rate due to the formation of a denser layer of Ag-MOFs on the surface.^56^ This result may suggest a limitation of the traditional Ag-MOF deposition approach compared to that proposed for M1. The reverse solute flux (*J*_s_) of both M1 and M2 membranes increased compared to the pristine membrane (Figure 5b). A larger *J*_s_ is to be expected to accompany the observed increase in water flux, as mass transport of water and salt usually increases or decreases simultaneously.^57^ Nevertheless, the change in reverse salt flux is more pronounced in the case of the M2 membrane, which displayed a significantly lower salt selectivity. The use of NMP to obtain a more uniform dispersion of Ag-MOFs in the procedure adopted for M2 may be responsible for the partial deterioration of the membrane, leading to defect formation at the support/active layer interface and easier solute transport.^34^ While the use of NMP was necessary to enable the functionalization of M2, this solvent was not required to obtain M1, whose preparation protocol is thus to be favored in terms of membrane transport performance.

The *J*_w_/*J*_s_ ratio, or reverse solute flux selectivity, includes the combined effect of membrane productivity and selectivity. It may be thought of as the volume of water permeated per mass of draw solute lost due to reverse transport. High values are indicative of membranes with desired transport performance. As evident in Figure 5c, M1 showed values of this ratio in line with those of the pristine membranes, which is an indication of uncompromised selectivity despite higher water flux. On the other hand, M2 displayed relatively reduced transport integrity, particularly due to poor solute selectivity, as outlined above.

### Antibacterial Properties of the Membranes

**Figure 6:**
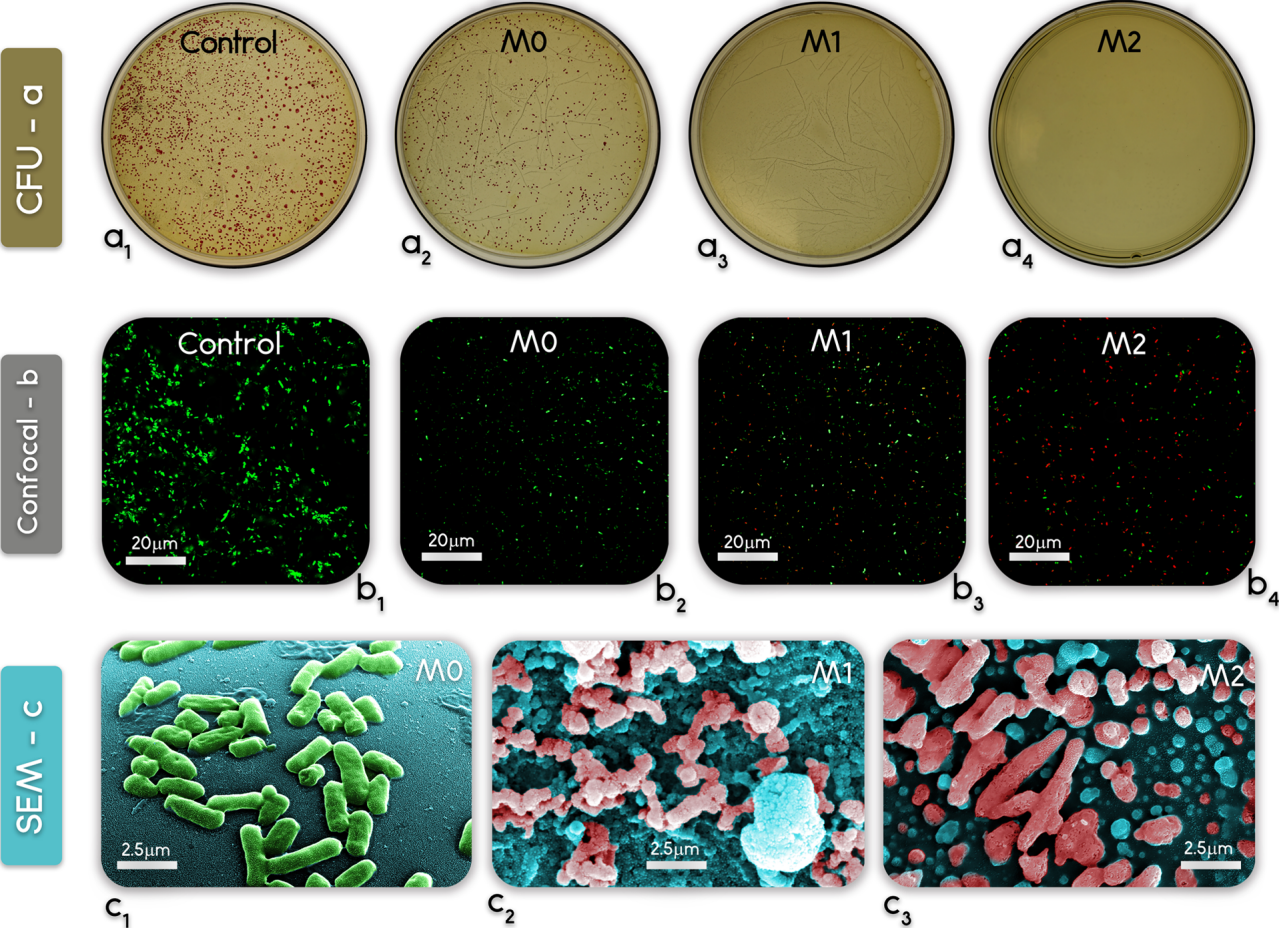
Results of antibacterial activity of the membranes against *E. coli*: (a_1–4_) heterotrophic plate count of cells suspended in the solution in contact with the sample surfaces, (b_1–4_) live/dead microscopy images of cells attached to the sample surfaces, and (c_1–3_) SEM images of bacteria upon contact with the membranes.

*E. coli* as model gram-negative bacteria was adopted to assess the antibacterial properties of the membranes. The representative confocal microscopies (Figure 6b_1_–b_4_) and plate count images (Figure 6a_1_–a_4_) provide a consistent rationalization of the results. For the M0 samples, no attached dead (red fluorescing) cells were observed under the microscope, and a large number of growing colonies are visible in Figure 6a_2_. The live-to-dead ratios of the attached cells were instead 1.38 and 0.31 for M1 and M2, corresponding to 42.0 and 76.3% of bacteria inhibition, respectively. The heterotrophic plate count of the unattached cells also suggested strong antibacterial activity by both M2 and M1 membranes with no viable cells grown on the plates, whereas M0 showed a growth of roughly 10^4^ CFU/mL, when the initial concentration in the control plate was 10^7^ CFU/mL. The SEM images (Figure 6c_1_–c_3_) also display healthy *E. coli* cells (Figure 6c_1_) on the M0 membrane and damaged *E. coli* cells upon contact or exposure to M1 and M2 membranes (Figure 6c_2_,c_3_). Ag-MOFs can kill or inactivate bacteria efficiently via different routes, including creating a breakthrough in the outer cell membrane followed by the leakage of cellular matters.^52^ The higher content of silver ions along with a more uniform distribution of Ag-MOFs on the M2 surface increased the probability that *E. coli* cells had lengthy and more direct exposure to silver ions compared to M1, thus explaining the higher antimicrobial activity observed for M2 samples.^30^ While being more cumbersome, the traditional surface modification procedure involving the deposition of Ag-MOFs onto the M2 membrane apparently allowed achievement of better intrinsic antimicrobial properties, consistent with the results from surface characterizations.

The antibacterial capability of these membranes may not be solely attributed to the presence of silver. Imidazole and its derivatives have also shown antiviral, antibacterial, and antifungal effects.^58,59^ In a recent study conducted to evaluate the antibacterial activity of three imidazole-based Ag-containing MOFs, Ag-2 methylimidazole demonstrated the highest antibacterial activity, owing to its high silver content and unique nanocrystal structure with sharp edges that can provide better interaction with bacteria.^35^ Please see further information in Figure S4 of the Supporting Information. In summary, the possible antibacterial mechanisms that may be present by application of the MOFs investigated in this study are related to (i) the gradual release of Ag^+^, with antibacterial properties likely proportional to the kinetics of release,^60^ (ii) the intrinsic properties of the Ag-2 methylimidazole organic linker, which is itself characterized by antimicrobial properties due to the imidazole heterocycle, and (iii) the crystal size and morphology of the nanostructures, particularly when they are in the order of tens to hundreds of nanometers, which may rupture the bacterial cells.^35,61^

### Flux Decline due to Fouling and Biofouling

**Figure 7:**
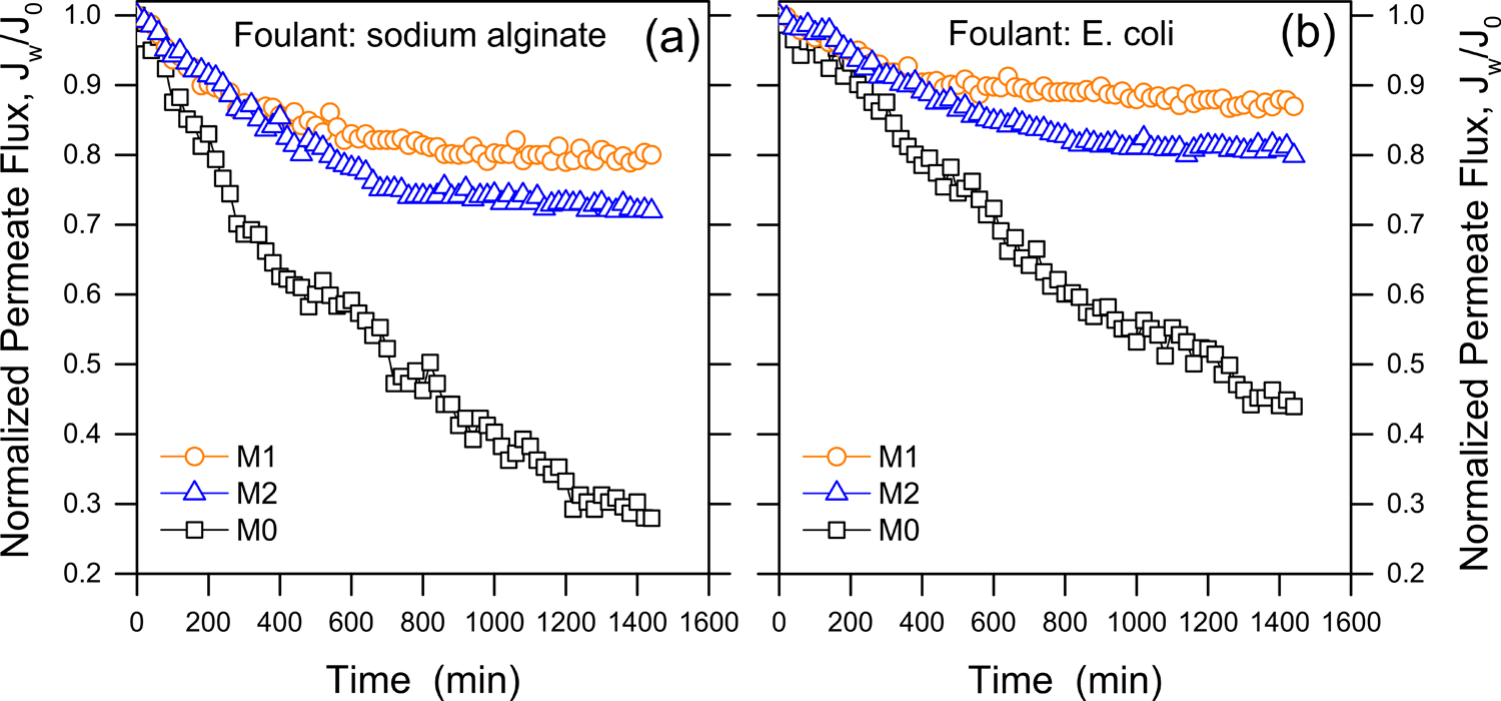
Antifouling performance of the pristine and of the surface-modified membranes during FO filtration tests. The model foulants were (a) sodium alginate at an initial concentration of 250 mg/L and (b) *E. coli* at an initial concentration of 10^7^ CFU/L. All the points are the average of two experiments.

Fouling experiments were conducted using *E. coli* as a model biofoulant and SA as an organic foulant. Upon the introduction of SA into the feed solution (Figure 7a), the water flux for all the membranes declined during the first few hours of operation although with a much steeper slope for the pristine M0 membranes. The flux decline ceased altogether for M1 and M2 membranes after roughly 7 h. The results of the biofouling experiments (Figure 7b) also suggest a significant improvement in terms of biofouling-induced flux decline for the functionalized membranes. Specifically, surface-functionalized membranes M1 and M2 maintained 80 and 72% of the initial flux in the organic fouling tests, and 87 and 80% in the biofouling tests. These figures are compared to respective fluxes that were 28 and 44% of the initial flux for the pristine membrane, indicating significant fouling mitigation brought about by surface modification. Please note that during the tests, the nominal driving force decreased by approximately 15–20% for all membranes due the combined effect of bulk DS dilution and reverse salt permeation. Also, the concentration of foulants in the FS increased up to roughly 25–30% for the pristine membrane and up to ∼40% at the end of tests involving the functionalized membranes due to the different rates of water permeation. The low flux decline for M1 and M2 membranes was thus a significant achievement, considering these experimental conditions.

These promising results are ascribed to both higher hydrophilicity^62^ and to the activity of silver that inactivated bacteria efficiently. Theoretically, the membrane with a higher surface loading of silver, i.e., M2, should show a better anti-biofouling performance. However, for both foulants (SA and *E. coli*), M1 membranes displayed a better resistance in terms of flux behavior. Since the hydrophilicity and surface potential of the two functionalized membranes were similar, the difference in fouling behavior may be partly rationalized with a less dominant effect of zwitterions on the M2 surface as a result of the relatively excessive loading of Ag-MOFs. Higher accessibility of zwitterions to the membrane surface would increase the density of the hydration layer, which decreases the chance of bacterial attachment. Also, the larger reverse flux of NaCl in M2 (see Figure 5) may give rise to a higher local ionic strength at the membrane/feed interface, facilitating more rapid foulant and bacteria attachment. Table 1 summarizes the different approaches and properties of FO membranes functionalized with silver-based or zwitterionic materials for biofouling mitigation.

**Table 1:** A Comparison of Different Strategies Applied for Antibacterial Functionalization of FO Membranes

substrate material	anti-biofoulant agent	modification approach	biofoulant	key feature of functionalization	ref.
PA-TFC	Ag-MOFs on zwitterionic coating	in situ growth exploiting zwitterionic binding sites	*E. coli*	facile, streamlined approach (especially for M1)	this work
				quick reaction time in room temperature with no dangerous solvents	
				excellent increase in hydrophilicity (50% reduction in contact angle)	
				no detrimental effect on membrane transport parameters (for M1)	
				substantial antibacterial activity (42–76% inactivation)	
				(bio)fouling mitigation during a 24 h operation without physical cleaning (87% flux retention throughout the biofouling test)	
					
CTA	Ag NPs regenerated by TiO_2_	in situ growth	adenosine triphosphate	moderately enhanced hydrophilicity	Nguyen et al., 2014^63^
				increased roughness	
				effective inhibition of bacterial growth	
					
PA-TFC	Ag-GO nanocomposite	click chemistry reaction	*E. coli*	super-hydrophilic properties	Soroush et al., 2015^5^
				significant bacterial activity reduction due to the synergetic effect of the Ag-GO nanocomposite	
				no adverse effect on the membrane transport properties	
					
PA-TFC	Ag-GO nanocomposite	EDC/NHS coupling and in situ reduction	*E. coli* and *E. faecalis*	enhanced silver loading and stability due to the GO presence	Soroush et al., 2016^64^
				increased surface hydrophilicity	
				98% antibacterial activity	
				75% antibacterial activity after regeneration	
					
PAN	Ag NPs	in situ reduction	*E. coli*	high antimicrobial activity for 14 days under laboratory conditions	Liu et al., 2015^65^
					
PA-TFC	Ag NPs on PDA coating	in situ growth	*E. coli* and *S. aureus*	enhanced hydrophilicity (contact angle of 40.6°)	Liu and Hu, 2016^32^
				increased roughness	
				strong antibacterial properties against *E. coli*	
					
PA-TFC	Ag-GO nanocomposite	EDC/NHS coupling	*P. aeruginosa*	slight reduction in surface roughness	Faria et al., 2017^66^
				significant decrease in bacterial attachment and viability	
				30% water flux decline during dynamic biofouling tests	
					
PA-TFC	Ag NP zwitterionic nanocomposite	ATRP grafting	synthetic wastewater supplemented with *P. aeruginosa*	smoother membrane surface	Liu et al., 2017^25^
				remarkable increased hydrophilicity (contact angle of 21°)	
				95% antibacterial activity	
				46% increase in dead cell biovolume	
				60% decrease in EPS content	
				8% water flux decline	
					
PA-TFC	Silica NP zwitterionic nanocomposite	ATRP grafting	*E. coli*	high surface hydrophilicity and reduced surface roughness	Liu et al., 2017^67^
				improved antifouling property	
				reduced water flux decline (17%)	
				increase in anti-biofouling resistance (96% reduction of the number of attached *E. coli*)	
					
PA-TFC	BSA-capped Ag NPs	grafting	*E. coli*	slight improvement in water permeability and salt rejection	Liu et al., 2017^68^
				low release rate and excellent stability during filtration	
				excellent antibacterial and high biofouling-resistant properties	
					
PA-TFC	Ag NPs on PDA coating	in situ reduction	synthetic wastewater supplemented with *P. aeruginosa*	improved hydrophilicity	Qi et al., 2018^69^
				good stability of Ag NPs and 96.1% antimicrobial activity after 24 h of cross-flow test	
				low water flux decline	
				proper antibacterial activity under both static and dynamic conditions	
				efficient biofouling mitigation during long-term operation	
					
PA-TFC	Ag NP zwitterionic nanocomposite	grafting and in situ reduction	*E. coli*	increased hydrophilicity, high water flux, and excellent selectivity	Qiu and He, 2018^70^
				simultaneous improvement of antiadhesive property	
				96% antibacterial activity	
				significant biofouling resistance and long-term anti-biofouling	
					
PA-TFC	Ag-MOFs	in situ growth	synthetic wastewater supplemented with *P. aeruginosa*	uniform distribution of Ag-MOFs on the PA layer	Seyedpour et al., 2019^11^
				irreversible binding of Ag-MOFs to the TFC surface	
				slight reduction in water permeability	
				nearly 100% antibacterial activity	
				high anti-biofouling performance	

### Release of Silver Ions

**Figure 8:**
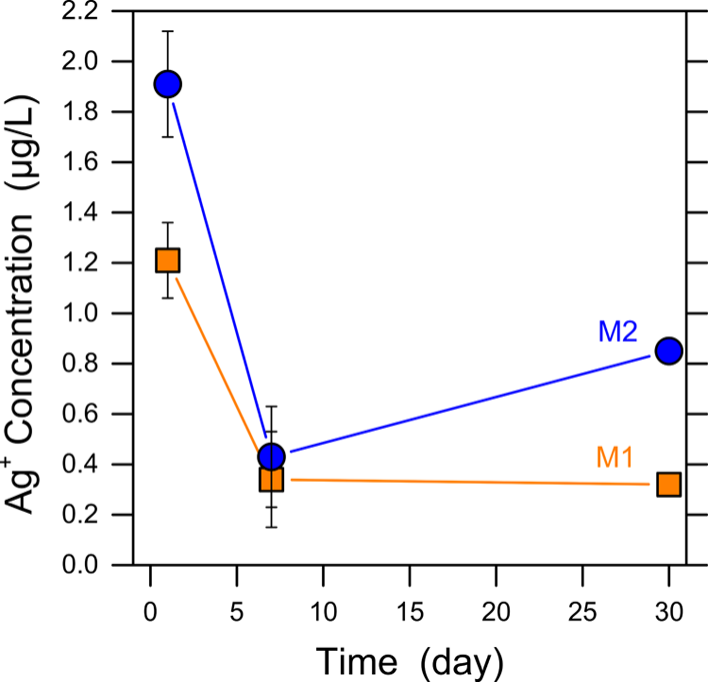
Results of silver ion leaching experiments from the MOF-modified membranes.

The stability of the antibacterial and anti-biofouling activity of the functionalized membranes significantly depends on the controlled release of silver.^1,37^ Therefore, the silver release rate was evaluated for the modified membranes. As can be observed in Figure 8, both membranes showed the same initial trend of reduction in the silver release rate in the first 7 days of monitoring. The M1 membrane exhibited a lower absolute release rate compared to M2 overall. This difference is, once again, consistent with the much more concentrated presence of Ag-MOFs on the M2 surface. As proposed above, oxygen-containing functional groups belonging to BPA may act as appropriate sites to retain silver ions, which ultimately leads to the stability of the Ag-MOFs and their binding to the membrane surface.^37^ Based on the data from the 30 day silver release experiment and on the typical tangential feed flow rate in FO and RO modules, the concentration of silver in the final concentrate stream of a cross-flow system would be <0.1 μg/L, well below maximum levels indicated by the WHO for drinking water quality (0.1 mg/L). While the overall depletion rate of silver ions was small for both functionalized membranes, the results imply that only growing Ag-MOFs in situ by exploiting interaction with underlying zwitterions (M1 membrane) can function as a more stable and durable Ag^+^ reservoir, capable of a controlled release to mitigate fouling and biofouling in a prolonged time period.

## Conclusions

This study evaluated the synergistic effect of silver-based MOFs and hydrophilic zwitterions to sustainably tackle fouling and biofouling in TFC membrane applications. Specifically, it proposed a streamlined approach to grow Ag-MOFs in situ on the membrane surface on a previously grafted layer of zwitterions comprising negatively-charged moieties that acted as binding sites for the silver metal (M1 samples). A more conventional approach was implemented where Ag-MOFs were separately synthesized and pre-deposited together with the zwitterions on the membrane surface before growing Ag-MOFs (M2 samples). The functionalization methods were successful as suggested by a combination of membrane characterization techniques. As a result, the modified FO membranes displayed suitable performance, with enhanced water flux in almost all cases.

Arguably, the most consequential results of this study concern the differences in functionalization and performance obtained with the two approaches, i.e., M1 vs. M2 membranes. A larger and more uniform amount of silver was made available on M2 membranes due to the pre-deposition of MOFs. Accordingly, while assays for viable bacteria suggested a substantial fraction of dead cells upon contact with both MOF-containing membranes, the M2 surface had better antibacterial properties compared to M1. On the other hand, its fouling behavior under filtration conditions was not better. Both M1 and M2 samples displayed similar and considerably lower flux decline compared to pristine membranes during the FO tests, when challenged with solutions containing alginate or *E. coli* bacteria. Specifically, the steady-state fluxes at the end of the fouling experiments decreased to a minimum of 70 and 80% of the initial flux for organic and biological fouling tests, respectively. The flux values for pristine membranes instead decreased to 28 and 44% of the initial flux for organic and biological fouling tests, respectively. Ag release experiments also revealed a relatively controlled release rate of silver, i.e., more stability of the functionalization especially for M1 samples. Overall, M1 showed clear advantages over M2 owing to (a) a more easily reproducible functionalization protocol, (b) yielding similar or improved results in terms of FO performance and fouling mitigation, (c) controlled silver leaching, and most importantly, d) presenting a more streamlined and potentially scalable approach toward achieving membranes with sustained antifouling and anti-biofouling properties.

## Supporting Information Available

ATR-FTIR spectra of the membranes; EDX elemental mapping of C, N, O, Br, and Ag elements on the two functionalized membranes; EDX spectra of the functionalized membranes; characterization of Ag-2MI nanocrystals (PDF)
