# Supplementary material for: In Situ Ag-MOF Growth on Pre-Grafted Zwitterions Imparts Outstanding Antifouling Properties to Forward Osmosis Membranes
Source: ACS Appl Mater Interfaces. 2020 Jul 17;12(32):36287–300. doi: 10.1021/acsami.0c12141 (PMC8009475; doi:10.1021/acsami.0c12141)
Supplement: Supplementary file 1 — am0c12141_si_001.pdf [file am0c12141_si_001.pdf]

Supporting Information for

In-Situ Ag-MOFs Growth on Pre-Grafted Zwitterions Imparts Outstanding Antifouling Properties to Forward Osmosis Membranes

Mehdi Pejman,[a,1] Mostafa Dadashi Firouzjaei,[b,1] Sadegh Aghapour Aktij,[c,d] Parnab Das,[b] Ehsan Zolghadr,[e] Hesam Jafarian,[f] Ahmad Arabi Shamsabadi,[g] Mark Elliott,[b,*] Mohtada Sadrzadeh,[c] Marco Sangermano,[h] Ahmad Rahimpour,[a,h,i,*] and Alberto Tiraferri[a,*]

[a] Department of Environment, Land and Infrastructure Engineering (DIATI), Politecnico di Torino, Corso Duca degli Abruzzi 24, 10129 Turin, Italy

[b]Department of Civil, Construction and Environmental Engineering, University of Alabama, Tuscaloosa 35487, USA

[c]Department of Mechanical Engineering, 10-367 Donadeo Innovation Center for Engineering, Advanced Water Research Lab (AWRL), University of Alberta, Edmonton, AB, T6G 1H9, Canada

[d]Department of Chemical & Materials Engineering, University of Alberta, Edmonton, AB T6G 1H9, Canada

[e]Department of Physics and Astronomy, University of Alabama, Tuscaloosa, AL, 35487, USA

[f]Department of Mining and Metallurgical Engineering, Amirkabir University of Technology, Tehran, Iran

[g] Department of Chemistry, University of Pennsylvania, Philadelphia, Pennsylvania 19104, United States

[h] Department of Applied Science and Technology, Politecnico di Torino, Corso Duca Degli Abruzzi 24, 10129, Turin, Italy

[i] Department of Chemical Engineering, Babol Noshirvani University of Technology, Shariati Avenue, Babol Mazandaran, 4714871167, Iran

[1]These authors contributed equally to this work

*Corresponding Authors:

Alberto Tiraferri. Email: alberto.tiraferri@polito.it

Ahmad Rahimpour. Email: ahmadrahimpour@nit.ac.ir

Mark Elliott. Email: melliott@eng.ua.edu

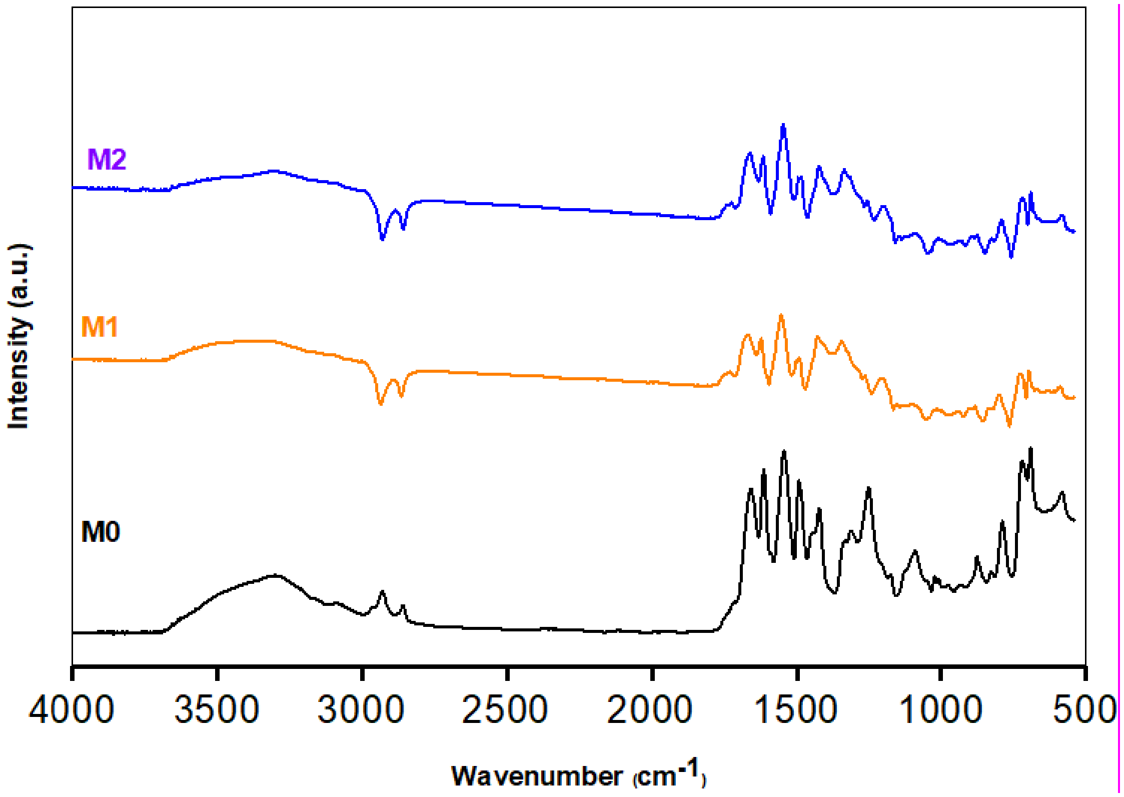

Figure S1. ATR-FTIR spectra of the membranes.

Membranes were characterized using ATR-FTIR spectroscopy to investigate their surface chemistry (**Fig. S1**). The broad peak observed at around 3300 cm^{-1} in all three spectra is attributed to O–H stretching.[1-3] The peak observed at approximately 1660 cm^{-1} is dominantly attributed to C=O stretching vibration of the amide, inherent of the polyamide layer of the membrane, in addition to C–C–N deformation vibration in the amide group.[3-4] The sharp peak at around 1544 cm^{-1} is assigned to C–N stretching and also N–H bending.[1, 5-6] This peak is a few wavenumbers shifted to the larger energy for M1 and M2 compared to M0, most likely due to the interactions caused by nanoparticles with the polyamide network in the modified membranes. Moreover, N–H stretching is observed at around 1610 cm^{-1}.[1, 5-6] The observed peak at 1249 cm^{-1} is attributed to C–O stretching.[4, 7] This signal almost disappeared in the spectra of modified

membranes presumably due to the presence of nanoparticles and occurring interactions. Additionally, several peaks in the range of 833-690 cm^{-1} likely correspond with rocking and bending vibrations of C–H.[7-8] A peak associated to C=O stretching of the carboxyl group is detected at 1730 cm^{-1}, which may be attributed to BPA, suggesting the presence of zwitterions on the surface of M1 and M2 membranes.[4, 9] The surface density of BPA was limited by the available carboxyl groups binding sites at the membrane surface, and to the yield of reaction between DEDA and BPA molecules, resulting in a relatively low intensity of this C=O peak, as proved by a previous study.[9]

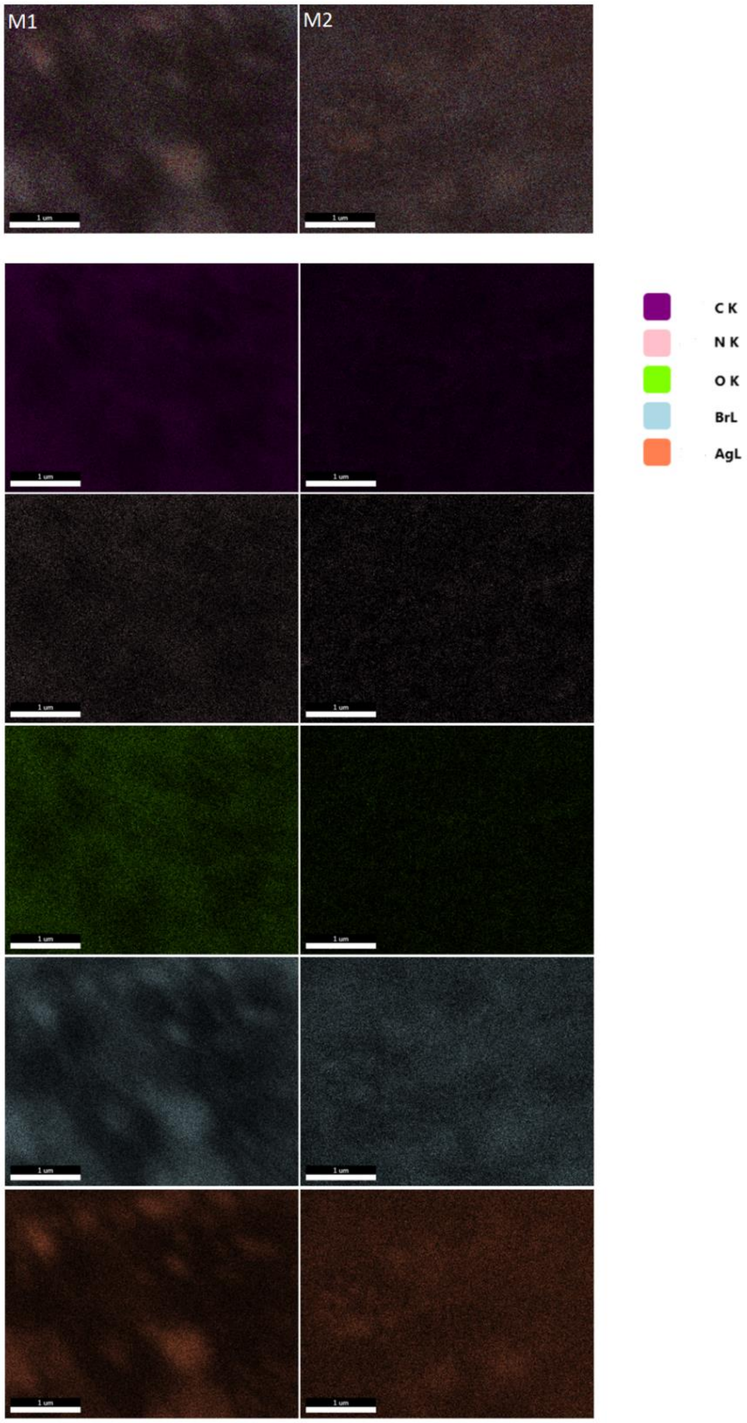

Figure S2. EDX elemental mapping of C, N, O, Br, and Ag elements on the two functionalized membranes.

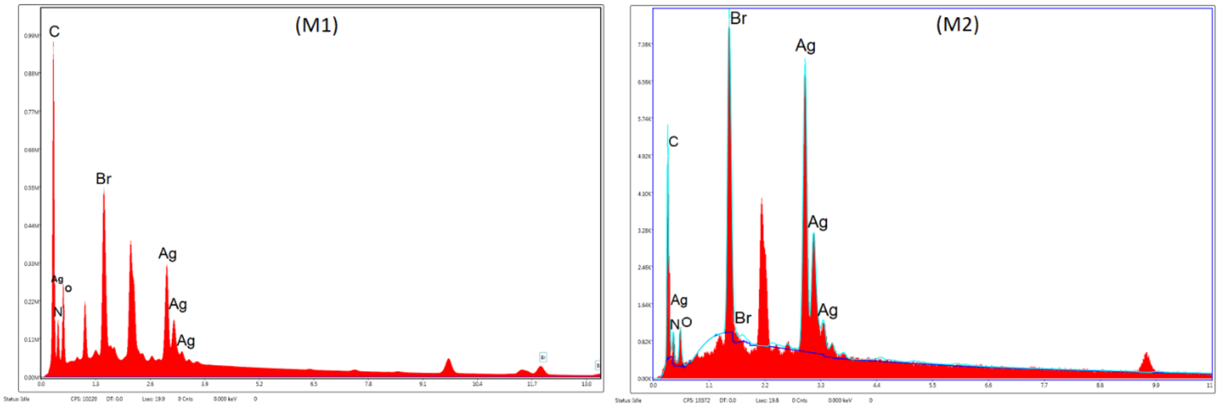

Figure S3. EDX spectra of the two functionalized membranes. Elements corresponding to the BPA zwitterions and to the Ag-MOFs are mentioned above their respective peaks.

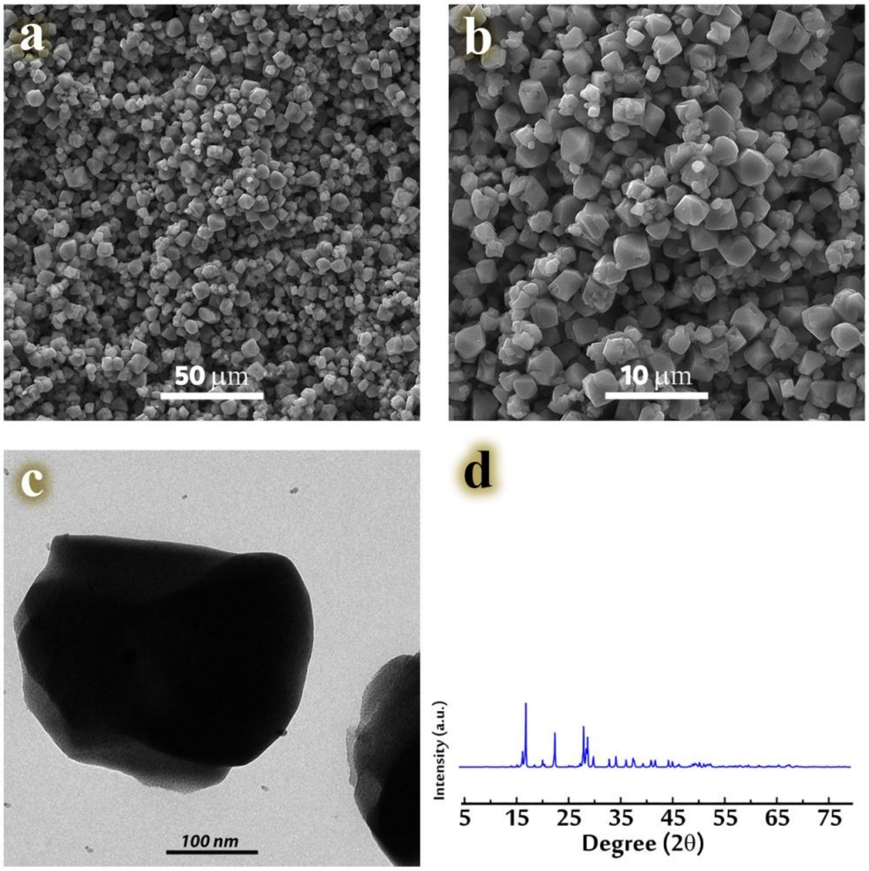

Figure S4. Characterization of Ag-2MI nanocrystals. (a and b) FE-SEM images at different magnifications, (c) TEM image, and (d) XRD spectrum corresponding the Ag-2MI structure. Data extracted from Seyedpour *et al.* (2020).[10]

Among various ligands used for supramolecular silver-based building blocks, multidentate building blocks containing a nitrogen (N) donor based on imidazole can lead to morphologically promising architectures. The Ag^+ ion has affinity to N-donor ligands and reacts with the supramolecular structure of these linkers. Representative FE-SEM and TEM images, as well an XRD spectrum of these structures, are reported above. These data are extracted from our published study on silver-based metal azolate frameworks, including Ag-2MI.[10]

References

1. Das, B.; Dash, S. K.; Mandal, D.; Ghosh, T.; Chattopadhyay, S.; Tripathy, S.; Das, S.; Dey, S. K.; Das, D.; Roy, S. Green Synthesized Silver Nanoparticles Destroy Multidrug Resistant Bacteria via Reactive Oxygen Species Mediated Membrane Damage. *Arab. J. Chem.* **2017,** *10* (6), 862-876.
2. Pinto, P. S.; Lanza, G. D.; Ardisson, J. D.; Lago, R. M. Controlled Dehydration of $Fe(OH)_3$ to Fe_2O_3: Developing Mesopores with Complexing Iron Species for the Adsorption of β-Lactam Antibiotics. *J. Braz. Chem. Soc.* **2019,** *30* (2), 310-317.
3. Yin, J.; Zhu, G.; Deng, B. Graphene Oxide (GO) Enhanced Polyamide (PA) Thin-film Nanocomposite (TFN) Membrane for Water Purification. *Desalination* **2016,** *379*, 93-101.
4. Tang, C. Y.; Kwon, Y. N.; Leckie, J. O. Effect of Membrane Chemistry and Coating Layer on Physiochemical Properties of Thin Film Composite Polyamide RO and NF Membranes. I. FTIR and XPS Characterization of Polyamide and Coating Layer Chemistry. *Desalination* **2009,** *242* (1-3), 149-167.
5. Baig, M. I.; Ingole, P. G.; Choi, W. K.; Jeon, J. d.; Jang, B.; Moon, J. H.; Lee, H. K. Synthesis and Characterization of Thin Film Nanocomposite Membranes Incorporated with Surface Functionalized Silicon Nanoparticles for Improved Water Vapor Permeation Performance. *Chem. Eng. J.* **2017,** *308*, 27-39.
6. Seyedpour, S. F.; Rahimpour, A.; Najafpour, G. Facile In-Situ Assembly of Silver-Based MOFs to Surface Functionalization of TFC Membrane: A Novel Approach toward Long-Lasting Biofouling Mitigation. *J. Membr. Sci.* **2019,** *573*, 257-269.
7. Wei, X.; Wang, Z.; Wang, J.; Wang, S. A Novel Method of Surface Modification to Polysulfone Ultrafiltration Membrane by Preadsorption of Citric Acid or Sodium Bisulfite. *Membr. Water Treat.* **2012,** *3* (1), 35-49.
8. Firouzjaei, M. D.; Shamsabadi, A. A.; Sharifian Gh, M.; Rahimpour, A.; Soroush, M. A Novel Nanocomposite with Superior Antibacterial Activity: A Silver-Based Metal Organic Framework Embellished with Graphene Oxide. *Adv. Mater. Interfaces* **2018,** *5* (11), 1701365.
9. Yi, M.; Hon, C. H.; Xion, S.; Wei, W.; Liao, R.-z.; Liang, S.; Lu, A.; Wang, Y. Zwitterion-Ag Complexes that Simultaneously Enhance Biofouling Resistance and Silver Binding Capability of Thin Film Composite Membranes. *ACS Appl. Mater. Interfaces* **2019,** *11* (17), 15698-15708.
10. Seyedpour, S. F.; Arabi Shamsabadi, A.; Khoshhal Salestan, S.; Dadashi Firouzjaei, M.; Sharifian Gh, M.; Rahimpour, A.; Akbari Afkhami, F.; Shirzad Kebria, M. R.; Elliott, M. A.; Tiraferri, A.; Sangermano, M.; Esfahani, M. R.; Soroush, M. Tailoring the Biocidal Activity of Novel Silver-Based Metal Azolate Frameworks. *ACS Sustain. Chem. Eng.* **2020,** *8* (20), 7588-7599.
